# Physical Activity and Metabolic Disorders—What Does Gut Microbiota Have to Do with It?

**DOI:** 10.3390/cimb47080630

**Published:** 2025-08-07

**Authors:** Aneta Sokal-Dembowska, Ewelina Polak-Szczybyło, Kacper Helma, Patrycja Musz, Maciej Setlik, Weronika Fic, Dawid Wachowiak, Sara Jarmakiewicz-Czaja

**Affiliations:** 1 Faculty of Health Sciences and Psychology, Collegium Medicum, University of Rzeszów, 35-959 Rzeszów, Poland; asokal@ur.edu.pl (A.S.-D.); ewpolak@ur.edu.pl (E.P.-S.); khelma@ur.edu.pl (K.H.); pm116471@stud.ur.edu.pl (P.M.); ms121668@stud.ur.edu.pl (M.S.); dw131242@stud.ur.edu.pl (D.W.); 2 Student Scientific Club of Human Nutrition, Faculty of Health Sciences and Psychology, Collegium Medicum, University of Rzeszów, ul. Warzywna 1a, 35-959 Rzeszów, Poland; wf130811@stud.ur.edu.pl

**Keywords:** physical activity, gut microbiota, metabolic diseases, obesity, diabetes mellitus, steatohepatitis associated with metabolic dysfunction

## Abstract

Obesity, type 2 diabetes mellitus (T2DM) and steatohepatitis associated with metabolic dysfunction (MASLD) are on the rise and pose serious health challenges worldwide. In recent years, researchers have gained a better understanding of the important role of the gut microbiota in the development and progression of these diseases. Intestinal dysbiosis can contribute to the occurrence of increased intestinal permeability, inflammation and reduced numbers of commensal bacteria. In obesity, these changes contribute to chronic low-grade inflammation and deregulated metabolism. In MASLD, gut microbiota dysbiosis can promote liver fibrosis and impair bile acid metabolism, while in T2DM, they are associated with impaired glycemic control and insulin resistance. Regular physical activity has a positive effect on the composition of the gut microbiota, increasing its diversity, modulating its metabolic functions, strengthening the intestinal barrier and reducing inflammation. These findings suggest that exercise and microbiota-targeted interventions may play an important role in the prevention and treatment of metabolic diseases.

## 1. Introduction

Metabolic disorders such as obesity, metabolic dysfunction-associated steatotic liver disease (MASLD), and type 2 diabetes mellitus (T2DM) are among the most prevalent and serious health challenges globally. Obesity, once considered a localized issue, has become a global epidemic, currently affecting nearly one in eight individuals worldwide. It significantly contributes to the development of insulin resistance, cardiovascular diseases and various cancers [1]. MASLD, a liver condition associated with metabolic syndrome, affects over one-third of the adult population and is linked to both obesity and T2DM [2]. T2DM alone affects nearly 830 million people worldwide with nearly 45% of individuals remaining undiagnosed [3].

The gut microbiota is a complex collection of bacteria, viruses, fungi, protozoa and archaea [4]. Metabolites produced by the gut microbiota play a crucial role in regulating human metabolism, particularly lipid and carbohydrate metabolism, and modulating immune responses and neuroendocrine regulation [5]. Acevedo-Roman et al. found that poor diet, frequent antibiotic use, intestinal inflammation, and oxidative stress can lead to gut dysbiosis, which is characterized by a loss of beneficial microbes and the excessive growth of harmful bacteria [6]. Gut dysbiosis has been linked to the development of obesity, MASLD and T2DM. In obesity, the dysbiosis of gut bacteria can lead to increased intestinal permeability, allowing bacterial toxins to enter the bloodstream. This may trigger low-grade inflammation and disrupt hormones that regulate appetite and satiety, such as Glucagon-Like Peptide-1 (GLP-1) and Peptide Tyrosine Tyrosine (PYY) [7,8]. In T2DM, dysbiosis has been linked to a reduction in beneficial bacteria and an increase in potentially harmful species, which can worsen blood glucose control and insulin sensitivity [9]. Similar imbalances in gut microbiota are observed in MASLD, where they can affect nutrient metabolism, increase intestinal permeability, and contribute to liver inflammation, potentially influencing disease progression [10].

Exercise has been shown to support gut health and improve metabolic functions. Evidence suggests that gut microbial diversity is more significantly influenced by a combination of aerobic and resistance training than by resistance training alone. Low-intensity exercise may offer only limited benefits in this regard. While moderate- to high-intensity activity is generally beneficial, excessive or prolonged high-intensity exercise may increase systemic inflammation [11]. Physical activity can also increase microbial diversity and elevate the numbers of helpful bacteria such as *Akkermansia*, *Faecalibacterium* and *Bifidobacterium* [11]. These microbes are important for producing short-chain fatty acids (SCFAs), regulating the immune system, and maintaining the intestinal barrier [12]. In individuals with MASLD, both aerobic and strength exercise have been associated with lower liver fat, improved insulin sensitivity, a healthier gut microbiota profile and decreased inflammation [13]. Similar benefits have been seen in T2DM, where exercise increases SCFA production and enhances blood sugar control—even in the absence of weight loss [14].

**Figure 1:**
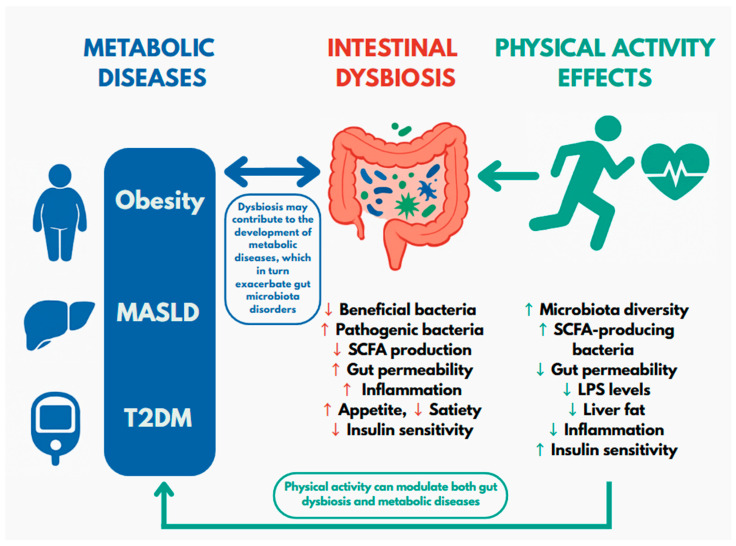
Relationships between gut microbiota, metabolic diseases and physical activity. MASLD—steatohepatitis associated with metabolic dysfunction; T2DM—type 2 diabetes mellitus; SCFA—short-chain fatty acids; LPS—lipopolysaccharide. The green arrows on the right indicate the beneficial effects of physical activity on both intestinal dysbiosis and metabolic diseases. This relationship is summarized in the figure in the note “Physical activity can modulate both intestinal dysbiosis and metabolic diseases.

The connection between the gut microbiota and metabolic diseases is becoming increasingly clear. Physical activity plays an important role not only in supporting overall health but also in promoting a balanced gut microbiota, making it a promising non-pharmacological strategy for the prevention and management of obesity, MASLD, and T2DM. The aim of this review is to summarize current scientific evidence on the role of the gut microbiota in metabolic diseases and to examine how physical activity influences gut microbiota, thereby affecting the development and progression of these conditions (Figure 1).

## 2. Obesity

In the 21st century, obesity has become one of the most serious public health problems, which, despite its global scale, remains neglected [15]. According to the World Health Organization (WHO), obesity is a complex, chronic disease caused by the excessive accumulation of adipose tissue in the body, which can negatively affect health [1]. According to the WHO data, the prevalence of obesity has increased significantly in both pace and scale [1,15]. Between 1990 and 2022, a two-fold increase in the number of adults with obesity was noted, as well as a four-fold increase in the number of youths with obesity. In 2022, it was estimated that one in eight people worldwide suffered from obesity [1]. In the period 1999–2023 in the United States, 19,451 deaths were caused by complications related to obesity among people aged 18–39 years [16]. Scientists predict that by 2050, the number of children and adolescents aged 5–24 in the United States with obesity will increase to 24 million and the number of adults aged 25 and over to 146 million [17]. The worldwide dynamic development of obesity generates huge financial outlays for medical care. In 2020, the financial costs associated with obesity amounted to 2.4% of the global gross domestic product. It is predicted that by 2035, these expenses will increase to USD 4.3 trillion [18]. In order to reduce the high costs of treating obesity, it is necessary to understand the causes of the disease, which will allow the selection of the best methods of prevention [18,19]. The etiology of obesity is extremely complex and includes biological, behavioral and environmental factors. Through their mutual influences, these factors create a state of imbalance between energy intake and energy expenditure, resulting in weight gain. Biological factors include genetics, prenatal conditions, neuroendocrine diseases, physical disabilities, menopause, medication, dysbiosis, the gut microbiome and past viral infections. Behavioral factors include a sedentary lifestyle, improper eating patterns, quitting smoking, excessive calorie intake and insufficient sleep. Environmental factors include culture, socioeconomic status, place of residence, environmental pollution, and the abundance of unhealthy food [19]. Obesity has many serious consequences for both physical and mental health. Excess body weight is a major factor increasing the risk of metabolic syndrome, which includes dyslipidemia, hypertension, hyperglycemia and atherosclerosis [20]. It also contributes to a higher probability of developing T2DM and insulin resistance [21]. Moreover, it increases the risk of heart rhythm disorders, obstructive sleep apnea (OSA), asthma, hypothyroidism or osteoarthritis [18]. In turn, among women with obesity, there is a higher risk of developing polycystic ovary syndrome (PCOS) and menstrual disorders, which leads to problems with conception and frequent miscarriages [22]. It is worth noting that obesity is the cause of about 4–8% of cancers. Moreover, it increases the risk of death from these cancers by about 17% [23]. Additionally, it should be mentioned that obesity affects mental health by increasing stress and contributing to the development of depression, eating disorders and body image perception disorders [24]. In order to improve the quality of life of people with obesity, comprehensive treatment should be implemented. One of the main pillars of therapy is dietary intervention, which aims to reduce body weight by changing eating habits [25]. Physical activity is helpful in the fight against obesity. When individually tailored, it can support weight loss and its maintenance. Regardless of weight reduction, regular exercise can mitigate some of the metabolic consequences of obesity [26]. Weight loss can also be achieved with supplementation or pharmacotherapy. The most effective drugs in the treatment of obesity are orlistat, bupropion, phentermine, lorcaserin, liraglutide, semaglutide and tirzepatide [27]. When the above-mentioned methods fail, the last resort for the patient is bariatric surgery. To undergo the procedure, the patient must meet specific criteria, e.g., a Body Mass Index (BMI) ≥ 40 kg/m^2^, BMI ≥ 35 kg/m^2^ with comorbidities that can be improved by weight loss, and BMI 30–34.9 kg/m^2^ with difficult-to-treat T2DM [25].

### 2.1. The Influence of Intestinal Dysbiosis on the Development of Obesity

There has been growing interest in microbiota in recent years [28]. They perform immunomodulatory and metabolic functions, maintain the integrity of the mucosa, and protect the host organism against pathogens. The gut microbiota are made up of 90% bacteria from the *Firmicutes* and *Bacteroidetes* classes. The remaining part is supplemented by the *Actinobacteria*, *Fusobacteria*, *Proteobacteria* and *Verrucomicrobia* classes, as well as viruses and fungi. The composition of microorganisms is individual and depends on, among others, body weight and lifestyle [29]. The gut microbiota profile of people with obesity is characterized by lower diversity and an increased ratio of *Firmicutes* to *Bacteroidetes* [30]. Additionally, in this group of individuals, an increase in the abundance of *Lactobacillus reuteri*, *Mollicutes*, *Fusobacteria*, and *Proteobacteria* and a decrease in the number of *Faecalibacterium prausnitzii*, *Methanobrevibacter smithii*, *Verrucomicrobia*, *Lactobacillus plantarum* and *paracasei* was noted [7]. The composition of the gut microbiota plays an important role in the development of obesity and its comorbidities by influencing the processes regulating energy utilization and nutrient absorption [31]. The gut microbiota obtain energy from the diet by metabolizing fiber that is undigested by the host. The substrates for this reaction are SCFAs, which provide about 10% of the daily energy requirement [32]. In obese individuals, higher amounts of SCFAs are found in the plasma and lower amounts in the feces [33]. Wang et al. used linear regression to examine the association between plasma SCFAs (butyrate/isobutyrate, isovalerate, and valerate) and the BMI and waist-to-height ratio (WHtR) in a cohort of Chinese adults. The researchers observed a positive correlation between plasma butyrate/isobutyrate levels and the BMI and isovalerate and total SCFA levels with the WHtR. Based on the results of the study, it can be concluded that higher SCFA values may have a potential impact on the development of obesity [34]. Increased SCFA synthesis in obese individuals is caused by a greater abundance of *Firmicutes* in their microbiota profile. This, in turn, leads to increased energy extraction from food by the body [35]. SCFAs are converted into energy during the citric acid cycle and mitochondrial β-oxidation in colonocytes. On the other hand, SCFAs not metabolized in colonocytes are transported via the portal circulation to the liver, where they act as substrates for the synthesis of glucose, long-chain fatty acids, and cholesterol [36]. Moreover, commensal bacteria are responsible for the production of choline, which is subsequently used for the synthesis of very-low-density lipoproteins [37]. The gut microbiota are also responsible for the metabolism of bile acids. Using the mechanisms of deconjugation and dehydroxylation, they convert primary fatty acids into secondary fatty acids. This action is due to the enzymes contained in bile salt hydrolase, which are present mainly in *Firmicutes* and *Bacteroidetes*. The lack of homeostasis in the intestinal microbial profile correlates with changes in the composition of secondary bile acids, which in turn results in increased fat absorption [38]. The gut microbiota play an important role in regulating lipid accumulation. Angiopoietin-like protein 4 (Fiaf) is responsible for regulating fatty acid oxidation and inhibits lipoprotein lipase (LPL) in adipose tissue. Certain components of the gut microbiota can suppress Fiaf expression, leading to increased lipid accumulation in adipose tissue [39]. In addition, commensal bacteria in a state of dysbiosis inhibit adenosine monophosphate kinase (AMPk) in the liver and muscles. This action results in a reduction in fatty acid oxidation, which implies the accumulation of fatty acids in these sites [40]. Glucagon-like peptide 1 (GLP-1) and peptide YY (PYY) are hormones secreted by L cells in the intestinal epithelium. They play an important role in the regulation of satiety and appetite [8]. Their release is mainly induced by SCFAs, which are ligands of free fatty acid receptors 2 and 3 (FFAR2/3) [41]. An imbalance between intestinal microorganisms can cause a disruption of intestinal hormonal homeostasis [40]. In people with excessive body weight, significant decreases in the amount of GLP-1 and PYY are observed, which directly affect changes in the regulation of satiety and appetite [8,40]. The gut microbiota demonstrate the ability to modulate low-grade inflammation (LGI), which plays a significant role in the development of obesity [40]. In the case of overweight people, higher concentrations of lipopolysaccharide (LPS) are observed [42], which are enterotoxins [43] found in the cell wall of Gram-negative microorganisms, e.g., *Bacterioides* [42]. The basic function of LPS is considered to be the modulation of the intestinal immune response [43]. A characteristic feature of obesity is a reduction in the abundance of *Bifidobacterium*, which compromises tight junction integrity and decreases the production of glucagon-like peptide 2 (GLP-2). As a result, intestinal membrane permeability increases, which allows LPS to be translocated from the intestinal lumen to the bloodstream [42]. In serum, LPS binds to LPS-binding protein (LBP), which enables the acquisition of LPS monomers via Cluster of Differentiation 14 (CD14). In subsequent stages, LPS is transferred by CD14 to myeloid differentiation protein-2 (MD-2) in the Toll-like receptor 4/Myeloid Differentiation Protein-2 (TLR4/MD-2). This is followed by TLR4 dimerization, which leads to the recruitment of the adaptor proteins myeloid differentiation primary response 88 (MyD88) and TIR-domain-containing adaptor-inducing interferon-β (TRIF). In the final stages of inflammation, MyD88 activates Mitogen-Activated Protein Kinases (MAPs) and the nuclear factor kappa-light-chain-enhancer of activated B cell (NF-κB) pathways, which induce the production of pro-inflammatory cytokines such as interleukin-6 (IL-6) and tumor necrosis factor α (TNF-α) [44].

### 2.2. The Influence of Physical Activity on the Gut Microbiota and Its Impact on Obesity

Physical activity is an important environmental factor that modulates the structure and metabolic functions of the microbiota [45]. Regular physical exercise increases the diversity of intestinal microorganisms mainly by influencing the *Bacteroidetes*-to-*Firmicutes* ratio, as shown by Mika et al. [46]. Moreover, Munukka et al., after a 6-week exercise intervention in a group of overweight women, noted an increase in the number of *Akkermansia* and a reduction in the abundance of *Proteobacteria* [47]. Quiroga et al. conducted observations in a group of children with obesity who participated in a 12-week training program. This study also shows a decrease in the number of the cluster *Proteobacteria* and classes *Betaproteobacteria* and *Gammaproteobacteria* and an increase in the abundance of classes *Actinobacteria*, *Clostridia*, and *Flavobacteriia*, and the genera *Blautia*, *Dialister*, and *Roseburia* [48].

Beyond its influence on the gut microbiota, physical activity also modulates systemic inflammation. Moderate-intensity exercise improves intestinal barrier integrity, limiting lipopolysaccharide (LPS) translocation into the bloodstream. This has been confirmed by Carbajo-Pescador et al. [49]. Additionally, exercise intensifies the production of carnosine, arabinose [50], and irisin [51], which inhibit the activation of the NF-κB pathway responsible for the production of Tumor Necrosis Factor alpha (TNFα), interleukin 1β (IL-1β), IL-6, monocyte chemotactic protein 1 (MCP-1) and chemoattractant keratinocytes (KCs) [50,51]. The anti-inflammatory effects of exercise have been confirmed in studies of overweight and obese adolescents after just two weeks of exercise. Reductions in inflammation and insulin resistance occurred regardless of changes in body weight [52]. Increased gut microbial diversity caused by regular physical exercise leads to weight loss, reduced inflammation, and improved metabolic function in individuals with obesity [53,54].

**Figure 2:**
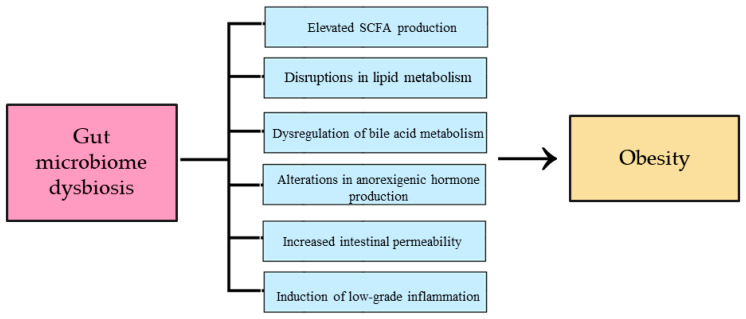
The impact of gut microbiome dysbiosis on obesity development. SCFA—short-chain fatty acids.

Of particular interest is *Akkermansia*, a genus consistently linked to physical activity and anti-obesity effects [47]. It regulates lipogenesis and fat metabolism, which limits the accumulation of adipose tissue. It also influences appetite and satiety mechanisms by modulating the hormones peptide YY (PYY) and glucagon-like peptide-1 (GLP-1) and enhancing insulin sensitivity. Additionally, it suppresses inflammatory activity by preventing increased intestinal permeability and LPS translocation, consequently limiting the production of pro-inflammatory cytokines [55]. Changes in the gut microbiota profile induced by exercise contribute to the balance of SCFA homeostasis [54]. Physical activity has been shown to increase SCFA levels in lean individuals [56]. Balanced SCFA concentrations regulate lipogenesis, promote fat oxidation, activate anorexigenic hormones, enhance insulin sensitivity, support the integrity of the intestinal barrier, and inhibit inflammation [54]. Recent studies have assessed the effect of SCFA supplementation on the development of obesity in a mouse model induced by a high-fat diet. The results of the analysis show a decrease in body weight and amelioration of inflammation in animals taking SCFA supplementation, which confirms their anti-obesity effect [57]. Despite the numerous benefits of physical activity, the mechanisms determining quantitative and qualitative changes in the gut microbiome profile are not yet fully understood. Knowledge gaps require further research into the impact of exercise on the microbiome and, consequently, obesity and its associated diseases [48]. The impact of intestinal microbiome dysbiosis on the development of obesity is shown in Figure 2.

## 3. Diabetes Mellitus

Diabetes mellitus (DM) belongs to a group of chronic diseases characterized by metabolic abnormalities. These include elevated serum glucose levels called hyperglycemia, elevated insulin levels called hyperinsulinemia, and lipid disorders [58]. Diabetes can be divided into several types: type I diabetes (T1DM), type II diabetes (T2DM), gestational diabetes, and other types of diabetes. However, the most common type is T2DM [59]. T2DM is largely caused by insulin resistance in body cells and progressive disorders in insulin secretion. The main function of insulin is to regulate the glucose concentration in the circulating blood. The β-cells of the pancreas, which are responsible for the production of insulin, begin to malfunction due to various disorders, resulting in the inability of body cells to use insulin properly. The result is a permanent increase in serum glucose levels [3,58]. However, it is not only pancreatic dysfunction that is a problem in the development of T2DM. The liver, brain, kidneys, adipose tissue, skeletal muscle, and small intestine, among others, also play important roles in the entire mechanism. Insulin resistance associated with diabetes increases glucose synthesis in the liver while reducing glucose uptake by adipose tissue, the liver, and muscle, thus leading to the further progression of the disease [60]. Analyzing diagnostic criteria in the diagnosis of this type of diabetes, we can distinguish four basic test results that should serve as a warning sign. The first is glycated hemoglobin (HbA1c), which reflects the average blood glucose level over the last 3 months. Its values equal to or greater than 6.5% may suggest existing abnormalities in our body. Fasting glucose concentration and glucose concentration in an oral glucose load test (OGTT) are also important parameters. A result of ≥126 mg/dL in the fasting glucose test and ≥200 mg/dL after a 2 h OGTT also indicate disorders related to glucose metabolism. The last parameter, glycemia ≥ 200 mg/dL accompanied by symptoms of hyperglycemia, may also indicate the presence of T2DM. In the diagnosis of diabetes mellitus, it is particularly important to make a correct diagnosis and determine the specific type of disease to determine the future prognosis and select appropriate treatments [3]. The complications of diabetes can be very extensive and can range from changes in smaller blood vessels (microvascular changes) to larger vessels such as the arteries (macrovascular changes). They most commonly affect the cardiovascular system, nervous system, visual system, urinary system, and skeletal system. Among microvascular complications, diabetic retinopathy and diabetic neuropathy are the most common. Macrovascular effects are much more dangerous to our health as they can cause serious changes in the functioning of our heart and brain [58].

Today, the number of diabetes cases worldwide is increasing every year. Furthermore, type II diabetes is the most commonly diagnosed form of disease and accounts for more than 90% of all global cases. Just a few years ago, the number of affected people was more than 422 million, while by 2022, the number was already close to 830 million. Interestingly, in low- and middle-income countries, the incidence of diabetes is increasing much faster compared to wealthier nations. In addition, the disease is increasingly being diagnosed in very young people. Until a dozen years ago, it was mainly the elderly who were associated with glycemic disorders, but over the years, the threshold for the disease has decreased significantly [3,58]. Also of concern is the fact that around 45% of diabetes cases worldwide remain undiagnosed. This means that almost one in two people with diabetes are unaware of their condition. Despite this, screening the population for diabetes is not currently recommended due to the low-cost effectiveness of such measures and the little scientific evidence supporting their effectiveness in improving patient health outcomes. However, special vigilance and control is recommended for high-risk individuals, those who are overweight and obese, have a positive family history of diabetes, or suffer from diseases associated with insulin resistance including polycystic ovary syndrome and gestational diabetes [61]. T2DM can be a disease with many complex backgrounds. Many researchers believe that the rapid economic development of countries, often coupled with unhealthy diets and a lack of physical activity, is responsible for its spread. Other risk factors that also play an important role in the pathogenesis of diabetes include excessive body weight, the use of stimulants, and occupational, environmental, and genetic factors [59,61]. Scientific evidence in recent years also points to the great role of the gut microbiota in the context of the occurrence of many chronic diseases, including T2DM [3].

### Diabetes Mellitus, Gut Microbiota, and Physical Activity

Increasingly, researchers are pointing to links between changes in the composition of the gut microbiota and diabetes. Intestinal dysbiosis may predispose individuals to the development of disrupted intestinal homeostasis, including abnormal intestinal barrier functioning, which may consequently increase inflammation in the body. Certain bacterial species (*Betaproteobacteria* and the ratio of *Bacteroidetes* to *Firmicutes*) have been linked to blood glucose levels [62]. Furthermore, Qin et al. suggest that the abundance of butyrate-producing bacteria in diabetic subjects is reduced, while the number of pathogenic or mucin-degrading bacteria is increased [9]. Gurung et al. point to *Bifidobacterium* and *Bacteroides* as protective factors in diabetes due to their beneficial effects in glucose metabolization [63]. Some confirmation of the effect of the gut microbiota on blood glucose levels is that transplanting the gut microbiota into patients diagnosed with metabolic syndrome resulted in increased tissue sensitivity to insulin [64]. Kootte et al. in their study also showed that transplanting the fecal microbiota (allogeneic) from lean individuals to people with metabolic syndrome can improve insulin sensitivity in the recipient, thus improving glucose metabolism [65]. Different results in this regard were obtained by Gómez-Pérez et al. [66]. Furthermore, *Blautia* bacteria have been linked to this, which may be related to abnormal glucose tolerance [67]. *Oscillibacter* and *Odoribacter*, on the other hand, have been associated with the occurrence of insulin resistance [68,69]. In a systematic review, Letchumanan et al., after including 18 studies, observed that levels of *Veillonella*, *Escherichia*, and *Collinsella* increase, while *Akkermansia muciniphila*, *Haemophilus*, and *Faecalibacterium prausnitzii* decrease, both in people before the diagnosis of diabetes and in those with newly diagnosed diabetes [70]. It is increasingly indicated that active metabolites of certain components may predispose individuals to T2DM. This is due to changes in intestinal epithelium permeability, altered energy metabolism in the host, and the presence of inflammation in the body [71]. The secreted lipids (LPSs) of bacteria are another factor that predisposes individuals to carbohydrate disorders due to an altered gut microbiome. Several studies have observed a higher LPS in the blood of diabetic patients compared to healthy individuals [72,73]. Such findings may suggest increased intestinal permeability in people with impaired carbohydrate metabolism [74]. Another factor exerting an effect on glucose metabolism is trimethylamine oxide (TMAO). This is a compound produced by a variety of intestinal bacteria as a result of the fermentation of dietary components. TMAO is also a factor that has pro-atherogenic properties [75]. Another element that affects reduced tissue sensitivity to insulin is bile acids (BAs). Some bacteria, for example, *Firmicutes*, *Bacteroidetes*, can regulate changes in BA; the most common effect involves altering the rate of the conversion of primary bile acids to secondary bile acids [76]. In numerous studies, the authors point to disorders that occur in the gut microbiota with a predisposition to insulin resistance, T2DM, and obesity [77,78,79,80]. The presence of metabolic diseases can be associated with a reduced quality of life, for example, by the constant monitoring of blood glucose levels, or difficulties performing simple tasks due to increased body weight. This in turn can predispose individuals to reduced self-esteem, anxiety, and depression [81,82]. Through, among other things, inappropriate lifestyles, including inadequate eating habits and a lack or low levels of physical activity, intestinal dysbiosis often coexists, which also contributes to the development of psychological disorders [83,84].

In addition to adopting a balanced diet to support the normal composition of the gut microbiota, it is also worthwhile to engage in regular physical activity. In patients with diabetes, exercise can reduce serum LPS and fecal zonulin levels, indicating a reduction in metabolic endotoxemia [12]. Aerobic exercise performed regularly at moderate and high intensities in overweight individuals may have a beneficial effect on reducing intestinal dysbiosis, which could also be considered in people with obesity and comorbid diabetes [56]. Aerobic exercise performed regularly at moderate and high intensities in overweight individuals may have a beneficial effect on reducing intestinal dysbiosis. This approach could also be considered in people with obesity and comorbid diabetes [85]. Regular exercise can also improve insulin resistance by up to 72 h [86]. Both resistance and aerobic training influence serum glucose levels and improve insulin action [87]. In addition to regulating carbohydrate metabolism, physical activity may also have a beneficial effect on reducing diabetes complications. Li et al. indicate that exercise alone reduces the risk of DM, while supplementation with *Lactobacillus caseii* may further reduce renal inflammation in DM by increasing SCFA production [14]. Lee et al. showed that regular aerobic activity can reduce levels of pro-inflammatory cytokines, which is also desirable for DM [88]. In another paper, the authors indicate that the abundance of *Akkermansia*, *Roseburia*, and *Faecalibacterium* increases after physical activity. These are bacteria that produce SCFAs that promote the maintenance of intestinal barrier homeostasis. They also suggest that exercise has the potential to reduce LPS-induced inflammation but empathize that further research is needed in this area [89]. Physical activity can increase the diversity of the gut microbiota, which in turn helps to promote an anti-inflammatory state, potentially affecting T1DM [90].

Patients with diabetes should be encouraged to engage in physical activity that is tailored to their individual capacity. Through regular exercise, the composition of the gut microbiota can change to a more favorable one, reducing inflammation by maintaining proper intestinal barrier function. This, in turn, can enhance tissue sensitivity to insulin and improve carbohydrate metabolism. It is also worth introducing proper eating habits to enhance the effect of physical activity on changes in the gut microbiota.

## 4. Metabolic Dysfunction-Associated Steatotic Liver Disease

MASLD is the new name for Non-Alcoholic Fatty Liver Disease (NAFLD). This new terminology emphasizes the important role of metabolic disorders in the development of this disease [91].

MASLD is described in the context of a multi-step pathogenesis model, known as the ‘multiple hit model’, in which the accumulation of fat in the liver is followed by other factors, such as genetic predisposition, environmental factors, microbiota disorders, and the development of insulin resistance, leading to disease progression [92]. MASLD is therefore defined as the presence of excessive hepatic triglyceride storage alongside at least one cardiometabolic risk factor (such as T2DM, obesity or hypertension) and in the absence of other identifiable causes of hepatic steatosis (such as excessive alcohol consumption) [93].

The term MASLD covers a broad spectrum of different liver conditions, including isolated steatohepatitis (MASL), steatohepatitis associated with metabolic dysfunction (MASH), fibrosis and cirrhosis [93].

Recent data show that the global prevalence of MASLD in adults exceeds 38%. Given the increasing prevalence of obesity and T2DM—two major risk factors for developing the disease—the proportion of people with MASLD is expected to steadily rise [2]. A meta-analysis conducted by Younossi et al., involving over two million adults with T2DM, showed that around 65% of those studied had MASLD. The researchers also found that the proportion of people with T2DM who had MASLD increased from around 56% to around 69% over the past three decades [2]. Predictive modeling by Le et al. showed that the prevalence of MASLD in the US adult population could rise from approximately 34% to over 41% by 2025 [94].

### 4.1. The Role of the Gut Microbiota in the Pathogenesis of MASLD

Given the well-documented impact of gut microbiota on human metabolism, abnormalities in its composition (i.e., gut dysbiosis) have been associated with an elevated incidence of obesity, dyslipidaemia, and T2DM. These conditions, in turn, increase the risk of developing MASLD [95].

A systematic review by Li et al., involving 1265 individuals, found that the gut microbiota of study subjects struggling with MASLD, as assessed from stool samples, was characterized by a higher abundance *of Escherichia*, *Prevotella* and *Streptococcus* bacteria, and a lower abundance of *Coprococcus*, *Faecalibacterium* and *Ruminococcus* bacteria, compared to that of healthy subjects [96]. Subsequently, Zazueta et al. noted that the incidence of MASLD was associated with changes in the abundance of gut bacterial populations involved in bile acid metabolism and SCFA production. The researchers also demonstrated that liver fibrosis was associated with lower levels of the gut bacteria *Ruminococcaceae UCG 013* and *Ruminoclostridium 6* and higher levels *of Sellimonas* [97].

Maslennikov et al. observed that an increase in the abundance of anaerobic bacteria belonging to the *Bacilli* class and the *Proteobacteria* phylum, alongside a decrease in the population of obligate anaerobes belonging to the *Clostridia* class (which help protect the intestinal barrier), may favor bacterial translocation. This, in turn, increases the risk of endotoxaemia. Pathogenic microorganisms that enter the liver via the portal vein can induce local inflammation. The chronic inflammation of the liver parenchyma promotes the development of a fibrotic process, consequently leading to liver fibrosis [10]. In a study by Li et al., liver fibrosis was induced in half of the animal models. It was also observed that the composition of the gut microbiota differed significantly between the diseased group and the control group. The researchers suggest that the gut microbiota could be used as a biomarker to help diagnose and determine the extent of liver fibrosis [98].

**Figure 3:**
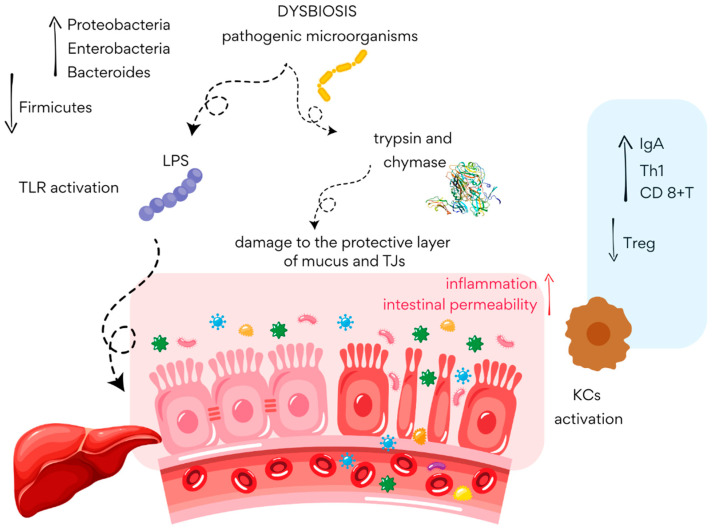
Disruption of intestinal barrier in MASLD (based on [99]). LPS—lipopolysaccharides; TJs—tight junctions; KCs—Kupffer cells; IgA—immunoglobulin A.

Additionally, intestinal barrier dysfunction, characterized by disrupted tight junctions (TJs) and increased intestinal epithelial permeability, plays a significant role in the development of MASLD [92]. Recent reports also highlight the importance of the gut vascular barrier (GVB). It has been shown that pathogenic microorganisms can downregulate the expression of TJs in both the epithelium and GVB, among others, by secreting proteases that lead to the degradation of these structures. As a result, the integrity of the mucosal barrier is disrupted, and local inflammation develops (Figure 3) [99].

### 4.2. Non-Pharmacological Treatment of MASLD

Currently, there is no effective pharmacological therapy for MASLD. However, the Mediterranean diet, characterized by a high intake of dietary fiber, is considered the gold standard for the prevention of steatohepatitis associated with metabolic disorders [100,101].

A study by Kazimierczak-Siedlecka involving 26 patients with MASLD showed that increasing dietary fiber significantly improved gut microbiota diversity [102]. For this reason, excluding highly processed foods from the diet and increasing dietary fiber intake is considered an effective method of improving gut microbiota composition and reducing metabolic disorder occurrence [103].

Furthermore, the beneficial effects of physical activity (especially resistance exercise) are observed in terms of both improved body composition and metabolic health, including a reduction in insulin resistance, which is important for the prevention and treatment of MASLD [104].

### 4.3. The Role of Physical Activity in Modulating Gut Microbiota and the Risk of Developing MASLD

Alongside diet, physical activity is an important environmental factor that influences the composition of the gut microbiota. Its type and duration can induce both quantitative and qualitative changes in the gut microbial community [105]. A sedentary lifestyle can lead to Triglycerides (TG) accumulation in the liver, IR, and the increased production of Reactive Oxygen Species (ROS), thereby leading to increased inflammation and fibrosis in the liver. However, physical activity appears to be able to reverse many of the adverse metabolic changes induced by this lifestyle [106].

It is likely that exercise improves the synthesis of gut metabolites by changing the gut microbiota [107]. Scheiman et al. observed an increase in the relative abundance of *Veillonella* bacteria in marathon runners. These microorganisms use lactate as their sole source of energy. Moreover, each gene involved in the major metabolic pathway that converts lactate to propionate showed higher relative abundance after exercise [108].

Studies in animal models provide robust evidence of an increase in *Akkermansia muciniphila* after moderate aerobic exercise [109]. Other bacteria that are more frequently observed in the gut microbiota of physically active people include *Faecalibacterium prausnitzii*, *Bifidobacterium* spp., *Lactobacillus* spp., *Prevotella* spp., and *Ruminococcus* spp. [11].

Patients with MASLD and advanced liver fibrosis show a reduced abundance of *F. prausnitzii*, the bacterium responsible for producing butyrate. This suggests a potential link between the deficiency of this bacterium and the severity of fibrotic processes. However, preclinical studies in mouse models have not shown supplementation with *F. prausnitzii* to lead to an improvement in parameters of diet-induced liver disease. Nevertheless, supplementation with sodium butyrate was found to improve hepatic steatosis in mice on a high-fat diet [110]. By contrast, a study by Yang et al. found that supplying *F. prausnitzii* reduced weight gain, liver and fat mass, and dietary energy intake while improving lipid and glucose metabolism in the liver and adipose tissue. Furthermore, reductions in low-grade inflammation and IR, as well as improvements in liver and intestinal barrier function, were observed [111]. This effect appears to be crucial in managing the metabolic disturbances associated with MASLD.

The results for *Ruminococcus* and *Prevotella* species are inconclusive. Some data suggest an association between these species and liver fibrosis development [112], confirming the complex role of the gut microbiota in liver disease pathogenesis.

A lower abundance of *Bifidobacterium* has been linked to the pathophysiology of liver diseases (MASLD, MASH, ALD, cirrhosis and HCC). Supplying *Bifidobacterium* has been associated with suppressing inflammation, increasing SCFA production, and enhancing intestinal barrier integrity [113]. Similarly, *Lactobacillus* spp. has been shown to have a beneficial effect in alleviating the course of MASLD. Various strains of *Lactobacillus* can produce SCFAs (such as butyrate, propionate and acetate), which play a vital role in maintaining intestinal homeostasis and increasing mucus production. SCFA production supports the restoration of the gut microbiota and contributes to restoring intestinal barrier integrity by enhancing the expression of tight junction proteins. Furthermore, *Lactobacillus* spp. modulates the immune response by promoting the production of anti-inflammatory cytokines (IL-10 and IFN-γ) while reducing the production of pro-inflammatory cytokines (TNF-α and IL-6). They also inhibit the colonization and growth of pathogenic bacteria and may reduce systemic inflammation by modulating the NF-κB signaling pathway [114].

### 4.4. The Influence of Different Types of Physical Activity on the Microbiota in MASLD

Csader et al. showed that, in patients with MASLD, 12 weeks of high-intensity interval training (HIIT) affected changes in gut bacterial interactions, but not the α- and β-diversity of the gut microbiota. There were also improvements in clinical parameters, including waist circumference, the BMI and insulin, HbA1c, and TG levels [115]. In a study by Babu et al., HIIT was found to reduce waist circumference and significantly decrease glucose levels in patients with MASLD while also increasing maximal oxygen uptake. Furthermore, changes in amino acid, lipid and bile acid metabolism were noted, which may confirm the positive effect of such physical activity on gut microbiota diversity. Reduced levels of bile acids have been observed in adipose tissue and urine [13], while elevated levels of circulating bile acids have been found in patients with MASLD [116].

Twelve weeks of HIIT combined with strength training and calorie restriction in people with obesity led to a decrease in liver enzymes (AST, ALT and GGT) and a significant increase in the relative abundance of commensal bacteria, such as *Akkermansia muciniphila*, *Parabacteroides merdae* and *Phocaeicola vulgatus*. [117]. In a study by Kazeminasab et al., the use of probiotic therapy containing strains of *Lactobacillus* (including *L. acidophilus*, *L. rhamnosus*, *L. casei*, *L. plantarum*, *L. salivarius*, and *L. paracasei*), *Bifidobacterium* (including *B. longum*, *B. breve*, *B. animalis*, and *B. bifidum*), *Enterococcus faecium*, *Lactococcus* spp., *Propionibacterium* spp. and *Acetobacter* in MASLD patients (with overweight or obese), combined with regular physical activity (walking, trotting, and running for ≥30 min, 3–5 times per week), led to a significant reduction in AST, GGT, LDL, TC and HOMA-IR compared to exercise intervention alone [118]. Therefore, in MASLD patients with increased body weight, the use of physical activity, caloric restriction and probiotic therapy may have a beneficial effect on improving metabolic parameters.

Kovynev et al. made an interesting observation in a study conducted on an animal model. It was shown that late exercise intervention at a more advanced stage of MASLD could have a significant therapeutic effect by influencing the gut microbiota. Introducing physical training (treadmill running; 1 h five times per week for 12 weeks) at this stage increased the abundance of SCFA-producing bacterial families and genera, such as *Akkermansia*, *Lachnospiraceae*, and *Rikenella.* Furthermore, transplanting microbiota into inactive mice led to a decrease in liver weight and plasma TG [119].

In the study by Cheng et al., it was found that both diet and physical activity increased the diversity and stability of the gut microbiota, either when used together or separately. It has also been demonstrated that the initial state of the gut microbiota is a key factor in determining the response to interventions. Therefore, it is crucial to develop personalized treatment strategies based on microbiota analysis [120]. Similarly, Calabrese et al. reported that a nutritional intervention involving the Mediterranean diet and physical activity had a positive effect on reducing the dysbiosis in patients with MASLD and increasing the resistance of the microbial communities inhabiting the gut [121].

**Figure 4:**
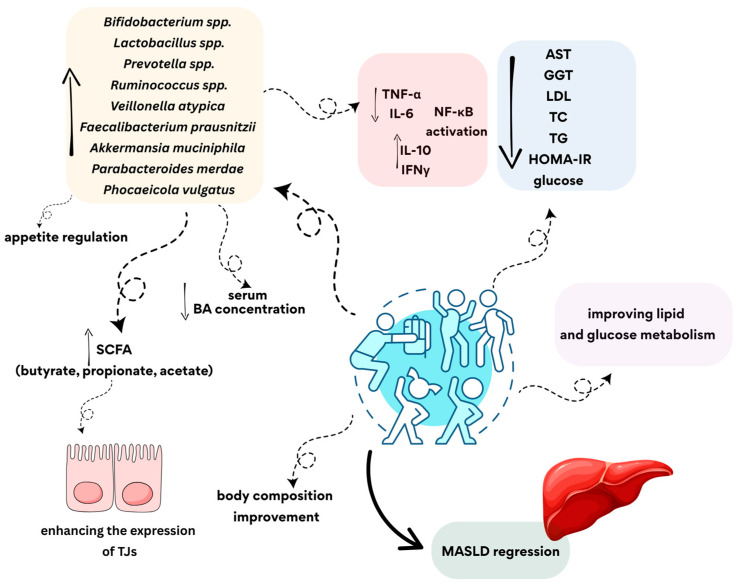
The potential impact of physical activity on alleviating symptoms of MASLD. SCFA—short-chain fatty acid; BA—bile acid; TJs—tight junctions; TNF-α—tumor necrosis factor alpha; IL-6—interleukin 6; IL-10—interleukin 10; IFNγ—interferon gamma; NF-κB—nuclear factor kappa-light-chain-enhancer of activated B cells; AST—aspartate aminotransferase; GGT—gamma-glutamyl transferase; LDL—low-density lipoprotein; TC—total cholesterol; TG—triglyceride; HOMA-IR—homeostasis model assessment of insulin resistance; MASLD—metabolic dysfunction-associated steatotic liver disease.

Physical activity plays an important role in modulating the gut microbiota of people with MASLD even in its advanced stages. This is associated with improved metabolic parameters, reduced liver inflammation, and improved gut barrier and liver function (Figure 4). However, the baseline composition of patients’ gut microbiota should be considered as this may affect the efficacy of the planned therapy.

## 5. Key Findings from the Review

The table below presents the key findings regarding the impact of physical activity on the intestinal microbiome and the risk of developing the diseases discussed in this review (Table 1).

## 6. Limitation of Available Scientific Data

Despite the growing evidence linking the gut microbiota to the development of metabolic diseases such as obesity, MASLD, and type 2 diabetes, much of this research is based on studies conducted in animal models. As is well known, such data do not fully reflect the complexity of the human microbiome and its interactions with the host. Furthermore, significant variability in microbiome composition among individuals, influenced by genetics, environment, and lifestyle, limits the generalizability of results and complicates the identification of microbial signatures.

Therefore, it is crucial to conduct longitudinal studies in humans to confirm cause-and-effect relationships and examine the long-term impact of physical activity and dietary quality on diseases such as obesity, diabetes, and MASLD. However, these studies should also consider the broader context of lifestyle and other factors that may influence the composition and function of the gut microbiota and contribute independently to the development of these diseases through different mechanisms.

## 7. Conclusions

Disruptions of the gut microbiota play an important role in the development and progression of metabolic diseases such as obesity, MASLD and T2DM. In obesity, gut microbiota dysbiosis is associated with increased dietary energy intake, chronic inflammation, and disruptions in appetite and metabolic regulation. In T2DM, dysbiosis is associated with impaired glycemic control and insulin resistance, and in MASLD, it contributes to liver fibrosis. Each of these diseases are also characterized by reduced microbial diversity and the loss of beneficial SCFA-producing bacteria. A common feature of these conditions is increased intestinal permeability, which facilitates the translocation of endotoxins into the bloodstream, promoting systemic inflammation and contributing to metabolic disorders. Regular physical activity has been shown to positively modulate the gut microbiota by increasing its diversity, promoting beneficial bacteria, strengthening the intestinal barrier and reducing inflammation, thereby contributing to improved metabolic health and better outcomes in obesity, T2DM, and MASLD. Exercise interventions can alter the *Bacteroidetes* to *Firmicutes* ratio, increase the abundance of *Akkermansia*, and reduce levels of *Proteobacteria*. In overweight and obese individuals, training programs increase populations of beneficial bacteria such as *Actinobacteria*, *Clostridia*, *Blautia*, *Dialister*, *Flavobacteriia* and *Roseburia*. In individuals with T2DM, exercise reduces serum LPS and fecal zonulin levels, enhances insulin sensitivity, and supports the growth of SCFA-producing and anti-inflammatory bacterial groups, including *Faecalibacterium*, *Akkermansia* and *Roseburia*. In MASLD patients, HIIT improves clinical markers including the BMI, HbA1c, insulin levels and liver enzymes while increasing the abundance of commensal bacteria such as *Akkermansia muciniphila*, *Parabacteroides merdae*, and *Phocaeicola vulgatus*. These findings underscore the potential of combining physical activity and microbiota-targeted interventions in the treatment of metabolic diseases.

## Figures and Tables

**Table 1:** Observed clinical outcome impact of physical activity.

Disease/Condition	Altered Gut Microbiota Taxa	Type of Physical Activity Studied	Observed Clinical Outcomes
Obesity	↑ *Bacteroidetes*, ↓ *Firmicutes* [46]	Aerobic activity	• gained weight • increase in lean body mass
	↑ *Akkermansia*, ↓ *Proteobacteria* [47]	Aerobic activity	• decrease in phospholipids and cholesterol in large VLDL particles • no changes in CRP • decrease in amine oxidase activity of pro-inflammatory VAP-1
	↓ *Proteobacteria*, *Betaproteobacteria*, *Gammaproteobacteria*, *↑ Actinobacteria*, *Clostridia*, *Flavobacteriia*, *Blautia*, *Dialister*, *Roseburia* [48]	Combined aerobic and resistance training	• inhibition of activation of obesity-associated NLRP3 pathway
	↑ *Parabacteroides*, *Bacteroides*, *Flavobacterium genera* ↓ *Blautia*, *Dysgonomonas*, *Porphyromonas* [49]	Combined aerobic and resistance training	• decreased HFD-induced body weight gain, metabolic syndrome and hepatic steatosis
	↑ *Bacteroides*, *Collinsella*, *Lachnospira* spp. ↓ *Faecalibacterium* spp. [56]	Resistance training	• decreased SCFA levels
Type 2 Diabetes	↑ *Verticillium* [122]	1.5-fold increase in physical activity	• improvement in fasting glucose levels • improvement in body composition • improvement in strength capacity induced by physical exercise
	↑ *Akkermansia*, *Roseburia*, *Faecalibacterium* [89]	Regular physical activity for 3 weeks	• production of SCFAs, which promote maintenance of intestinal barrier homeostasis
	↑ *Lachnospirales (Eubacteriales)*, *Enterococcus* spp., *Clostridium Cluster IV* [123]	combined aerobic and resistance moderate intensity continuous training	• butyrate producers
	↑ *Oscillospirales (R. bromii)* [123]	combined aerobic and resistance high-intensity interval training	• butyrate producers
MASLD (metabolic dysfunction-associated steatotic liver disease)	↑ *Faecalibacterium prausnitzii* [111]	Aerobic activity	• reduction in body weight, liver weight and fat mass • improvement in lipid and glucose metabolism in liver and adipose tissue • reduction in low-grade inflammation, reduction in IR, and improvement in liver and intestinal barrier function
	↑ *Bifidobacterium* [113]	Aerobic activity	• suppression of inflammation • increase in • SCFA production • strengthened integrity of the intestinal barrier
	↑ *Lactobacillus* spp. [114]	Aerobic activity	• synthesis of intestinal metabolites • maintenance of integrity of intestinal barrier • promotion of synthesis of anti-inflammatory cytokines, e.g., IL-10 and reduction in expression of pro-inflammatory cytokines TNF-α and IL-6 • modulation of NF-κB signaling pathway
	↑ *Akkermansia muciniphila*, *Parabacteroides merdae*, *Phocaeicola vulgatus* [117]	High-intensity interval training (HIIT)	• decrease in liver enzymes (AST, ALT and GGT)

VLDL—very-low-density lipoprotein; CRP—mC-reactive protein; VAP-1—Vascular Adhesion Protein-1; NLRP3—NOD-like receptor family, pyrin domain containing 3; HFD—high-fat diet; SCFA—short-chain fatty acid; IL—interleukin; TNF-α—tumor necrosis factor alpha; NF-κB—nuclear factor kappa-light-chain-enhancer of activated B cells; AST—aspartate aminotransferase; ALT—alanine aminotransferase; GGTP—gamma-glutamyl transferase, ↑—increase; ↓—decrease

## Data Availability

Not applicable.
